# Successful Conservative Management of Acute Appendicitis in a Coronavirus Disease 2019 (COVID-19) Patient

**DOI:** 10.7759/cureus.7834

**Published:** 2020-04-26

**Authors:** Kulachanya Suwanwongse, Nehad Shabarek

**Affiliations:** 1 Internal Medicine, Lincoln Medical Center, New York City, USA

**Keywords:** appendicitis, appendectomy, covid 19, coronavirus disease (covid-19), novel coronavirus

## Abstract

Appendectomy is the gold standard of treatment for acute appendicitis; however, recent evidence suggests conservative management with intravenous antibiotics may provide similar outcomes and can be used as an alternative in selected patients. Performing appendectomy in acute appendicitis patients with 2019 novel Coronavirus Disease (COVID-19) is challenging, as it involves considerable operative risks for the patients and risks for health care professionals (HCPs) exposed to COVID-19. Medical management eliminates the morbidity and mortality associated with surgery but involves significant risks of treatment failures that, in turn, may lead to perforation, peritonitis, and death. We are reporting a case of a middle-aged man with multiple co-morbidities, who was diagnosed with COVID-19 and acute appendicitis. Our patient received intravenous antibiotics for seven days with a significant improvement in symptoms. Our case report illustrates the implementation of successful conservative treatment for acute appendicitis in COVID-19 patients.

## Introduction

Acute appendicitis is the most common cause of surgical abdomen, with a lifetime prevalence of 7% [1]. Appendectomy is the gold standard of care for patients with acute appendicitis, but recent evidence showed that conservative treatment with intravenous antibiotics may provide similar outcomes [2]. Non-operative treatment for acute appendicitis has additional benefits, including the elimination of the mortality and morbidity risks associated with surgery, as well as the complications of anesthesia, infection, and bleeding, the obliteration of the long-term complications of abdominal surgery, such as bowel obstruction and chronic wound pain, a decrease in the treatment cost and the length of hospital stay [2-4].

Patients with the novel Coronavirus Disease 2019 (COVID-19) have substantially higher operative risks due to the compromise of lung function and the cytokine storms that result in systemic inflammatory response syndrome (SIRS) and multiple organ dysfunction. To date, there is no report regarding the outcomes of non-operative treatment for acute appendicitis in COVID-19 patients. We are presenting a case of successful conservative management of acute appendicitis in a COVID-19 patient to support the role of non-operative management for acute appendicitis in selected cases.

## Case presentation

**Figure 1:**
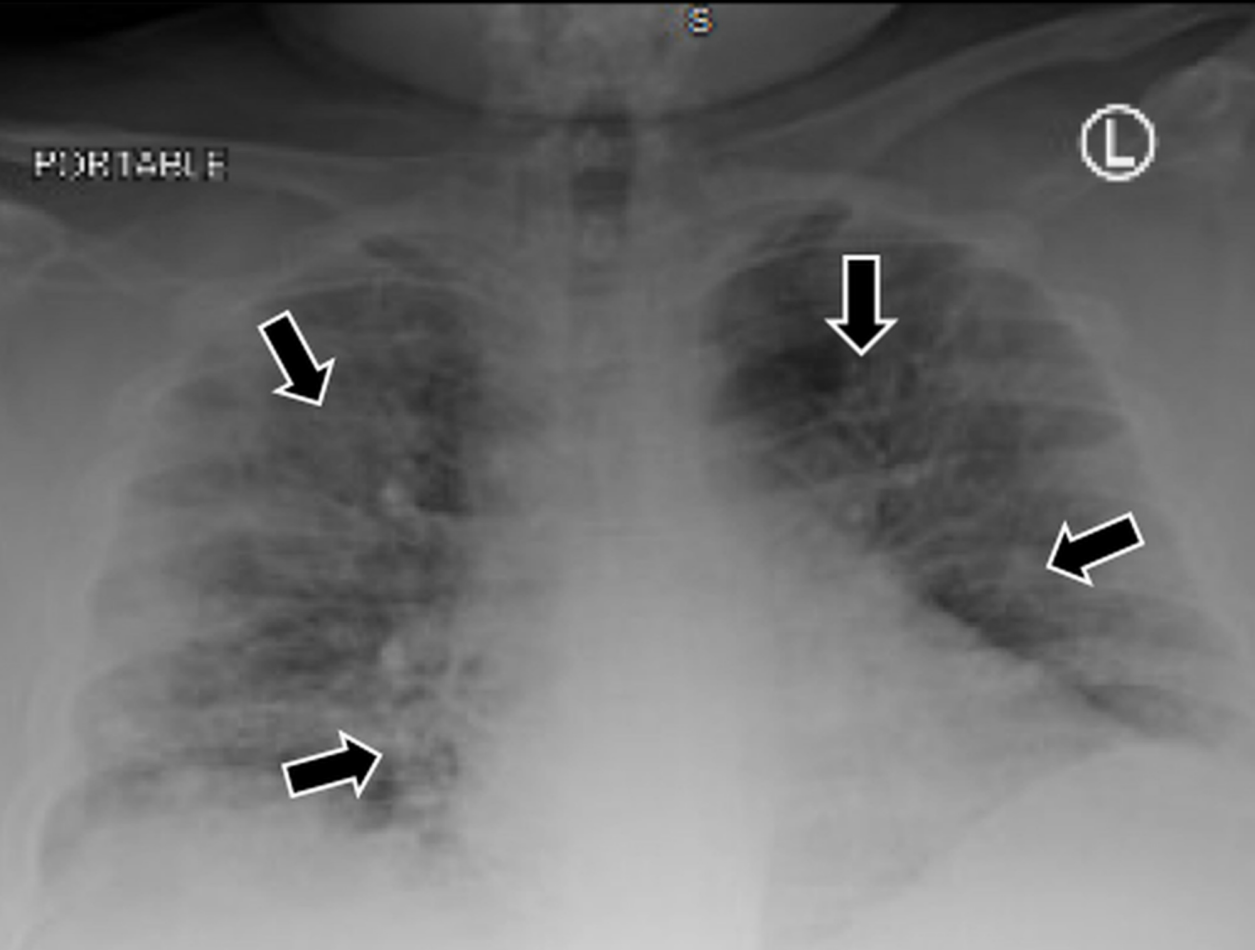
CXR showed bilateral multiple patchy infiltrates CXR: chest X-ray

A 47-year-old male presented to the emergency department with worsening generalized abdominal pain, which was aggravated by movement and associated with nausea and vomiting. He also reported fever, dry cough, and mild dyspnea. His past medical history included diabetes mellitus, hypertension, and morbid obesity with a body mass index (BMI) of 59.18. On initial evaluation, his vital signs were unremarkable. Oxygen saturation was 92% on room air. His lung exam was normal. The abdominal exam revealed generalized tenderness without guarding. His chest X-ray (CXR) showed bilateral multiple patchy infiltrates as demonstrated in Figure 1.

**Figure 2:**
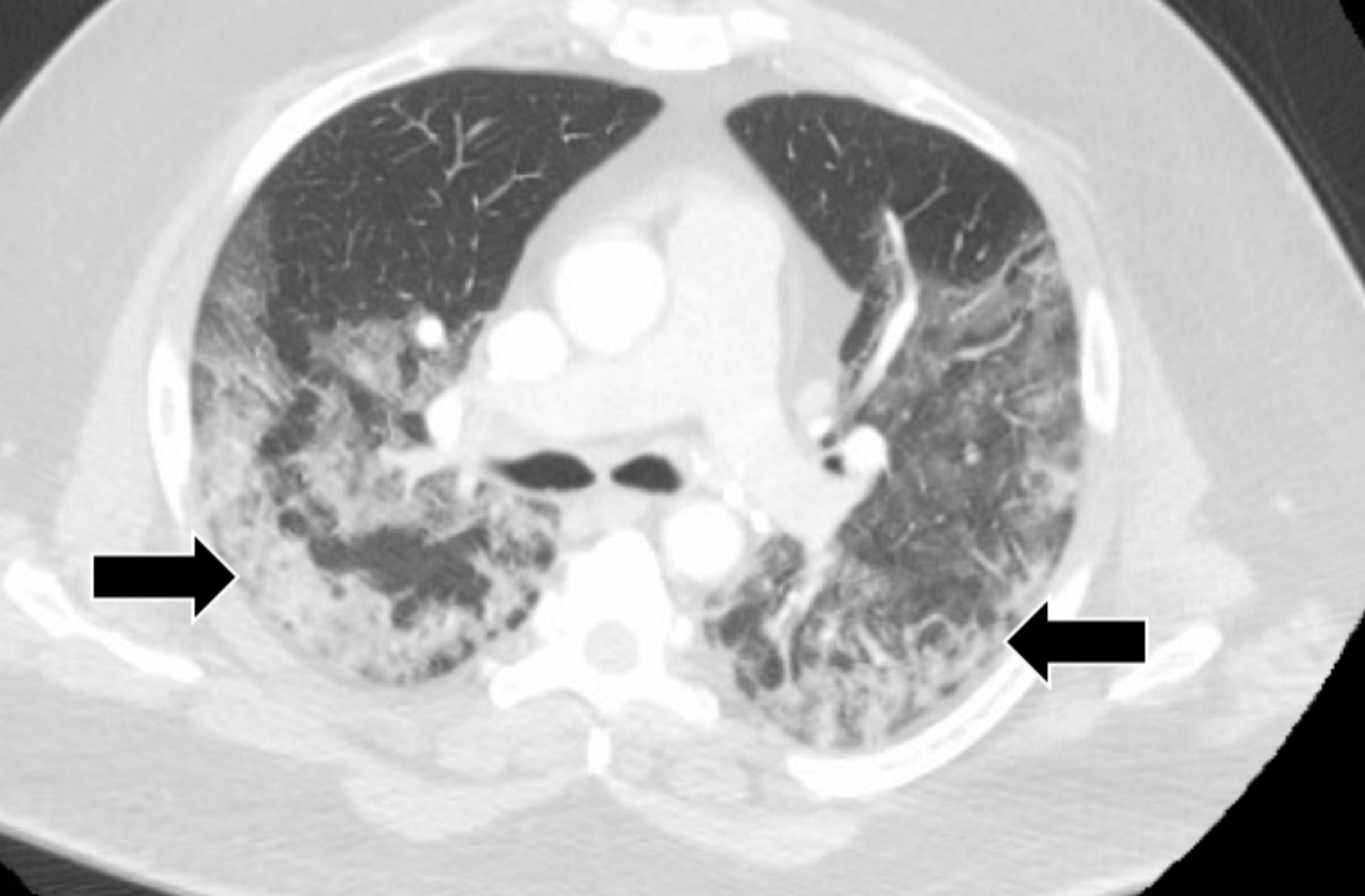
CT chest showed bilateral ground-glass opacification CT: computed tomography

A chest computed tomography (CT) scan revealed bilateral ground-glass opacification (Figure 2).

**Figure 3:**
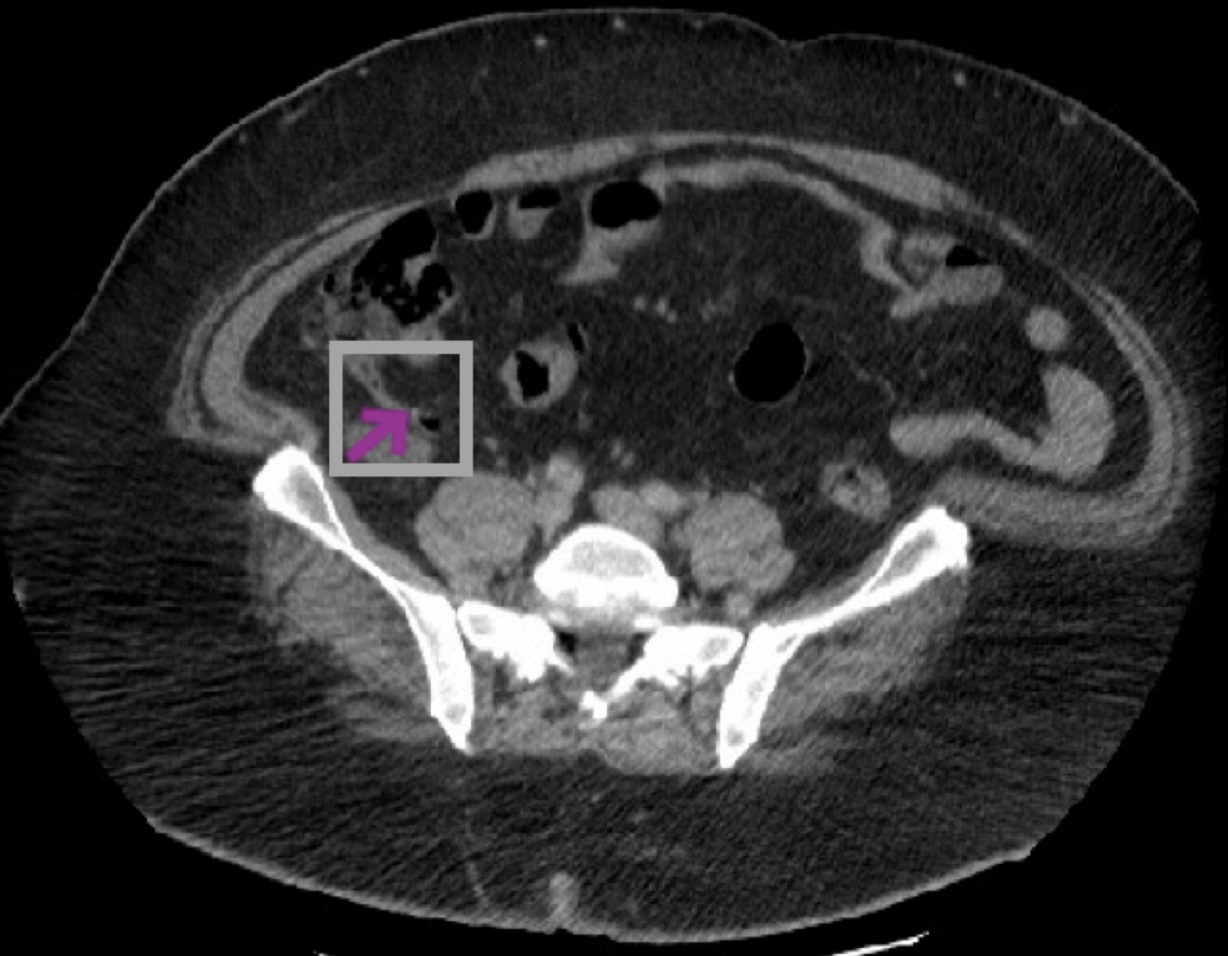
CT abdomen showed distended fluid-filled appendix CT: computed tomography

An abdomen CT was performed, which illustrated the dilation of fluid-filled appendix and infiltration of the surrounding fat, compatible with acute appendicitis (Figure 3).

Laboratory results were as follows: white blood count (WBC) 10.37 x10^3 ^cells per cubic millimeter with lymphocytes of 13.5%, C-reactive protein 11.55 milligrams per deciliter (mg/dl) (normal <0.4 mg/dl), ferritin 1376 nanograms per milliliter (ng/mL) (normal 30-400 ng/mL), D-dimer 356 ng/mL (normal <230 ng/ml), lactate dehydrogenase (LDH) 393 units per liter (U/L) (normal 135-225 U/L), and procalcitonin 0.17 ng/ml (normal: <0.09 ng/ml). His creatinine was significantly increased to 1.35 mg/dl as compared with his baseline at 0.83 mg/dl. His COVID-19 polymerase chain reaction test from the nasopharyngeal swab was positive.

He was admitted to the medicine floor due to COVID-19 pneumonia and acute appendicitis. Hydroxychloroquine and azithromycin were given for five days for the treatment of COVID-19. Due to the substantially high operative risks, the shared decision was made to provide medical management for his acute appendicitis. He received intravenous piperacillin/tazobactam 4.5 milligrams every six hours for seven days and his abdominal pain was resolved. He was discharged on hospital Day 8 without complications.

## Discussion

The diagnosis of acute appendicitis may be challenging in COVID-19 patients. Fever, nausea, vomiting, and abdominal pain may be mistaken as symptoms of COVID-19. In our patient, there was no sign of peritonitis on the abdominal exam, which was likely due to his morbid obesity. Acute appendicitis was diagnosed based on clinical signs but other investigations including ultrasound and CT scan of the abdomen are helpful in difficult cases. Misdiagnosis of acute appendicitis can lead to appendiceal perforation, appendiceal abscess, peritonitis, sepsis, and death. Clinicians should include acute appendicitis in the differential diagnosis of COVID-19 patients who report abdominal pain.

Although there is increasing evidence that intravenous antibiotics can be used as alternative management, especially in patients with a high operative risk, appendectomy remains the gold standard of care for patients with acute appendicitis. Surgical appendectomy provides curative treatment without a recurrence of appendicitis but associated with significant operative risks, post-surgical complications, higher costs, and longer recovery times. Conservative management is less invasive but associated with treatment failure and the chances of recurrences appendicitis [2-4].

COVID-19 patients have considerable operative risks as a result of direct lung injury and multiple organ dysfunctions. Our patient has multiple co-morbidities and had hypoxemia and acute kidney injury from COVID-19, which posed significantly high morbidity and mortality risks from an operation. Thus, the shared decision was made to initiate medical management with intravenous antibiotics, which successfully treated his conditions.

To date, there is a lack of good evidence, i.e., high-quality randomized controlled trials (RCTs) supporting the use of antibiotics in the treatment of COVID-19. Thus, the selection of antibiotics for the medical management of acute appendicitis in COVID-19 patients may not differ from the general population.

The non-operative management of acute appendicitis provides an additional advantage in terms of limiting the health care professionals' (HCPs') exposure to COVID-19, particularly in the setting of limiting personal protective equipment. Our case report suggests the role of conservative treatment of acute appendicitis in COVID-19 patients. However, more research is needed to evaluate the short- and long-term outcomes of the conservative treatment of acute appendicitis in patients with COVID-19.

## Conclusions

Intravenous antibiotics may be used as an alternative treatment of acute appendicitis in COVID-19 patients to eliminate operative risks and the risks of HCPs' exposure to COVID-19. To date, there is no high-quality evidence demonstrating the benefit of any antibiotics on COVID-19 treatment outcomes. Therefore, the optimal selection of antibiotics for the medical management of acute appendicitis in COVID-19 patients should be similar to that in patients not suffering from COVID-19.
